# The Post-Vaccination Quantitative Total Immunoglobulin Levels against SARS-CoV-2 in Healthcare Workers: A Multi-Centric Cohort Study in India

**DOI:** 10.3390/vaccines10091535

**Published:** 2022-09-15

**Authors:** Mangayarkarasi V. Babu, Dhrubajyoti J. Debnath, Mukesh Tripathi, Yalamanchili Samatha, Sumita Shankar, Vivekanand Kattimani, Dhanasekar Voloya Manikam, Pradeep Kumar

**Affiliations:** 1 All India Institute of Medical Sciences, Mangalagiri 522503, Andhra Pradesh, India; 2 SIBAR Institute of Dental Sciences, Guntur 522509, Andhra Pradesh, India; 3 Guntur Institute of Medical Sciences, Guntur 522004, Andhra Pradesh, India

**Keywords:** SARS-CoV-2, immune response, immunoglobulins, vaccination, antibodies, enzyme-linked immunosorbent assay, seroconversion

## Abstract

Healthcare workers (HCWs) in India received the AZD1222 and BBV152 vaccines from January 2021 onwards. The objective of this study was to compare the immune response (seropositivity rate and geometric mean titer (GMT), and 95% confidence interval (CI)] against severe acute respiratory syndrome coronavirus 2 (SARS-CoV-2) in HCWs who received these vaccines, after the first and second doses. Therefore, the total immunoglobulin (Ig) levels specific to SARS-CoV-2 were measured using quantitative enzyme-linked immunosorbent assay (ELISA). The study population of 133 HCWs consisted of two groups in which the immune response was measured for the AZD1222 and BBV152 vaccines. Data collection was performed from 6 February to 20 August 2021. Four weeks after the first and second dose, the odds ratio of seroconversion for AZD1222 and BBV152 vaccine was 10.3 times (95% CI: 4.5–23.7) and 15.9 times (95% CI: 6.3–39.9), respectively. The GMT was 6392.93 and 6398.82 U/mL for AZD1222 and 1480.47 and 990.38 U/mL for BBV152 after the first and second doses, respectively. Both vaccines elicited an immune response, but the seroconversion rate and GMT after each dose were significantly higher for AZD1222 than those for the BBV152 vaccine in this study.

## 1. Introduction

COVID-19 infection is a severe acute respiratory syndrome caused by severe acute respiratory syndrome coronavirus 2 (SARS-CoV-2) [1]. SARS-CoV-2 is a newly emergent coronavirus, first recognized in Wuhan, Hubei province, China, in December 2019. This is a positive-sense, single-stranded, highly contagious RNA virus that can be transmitted among humans. According to an announcement made by the Health Ministry of India, the COVID-19 vaccination drive started with two vaccines: AZD1222 and Bharat Biotech BBV152 vaccines, in January 2021. The two vaccine products were the non-replicating chimpanzee adenovirus vaccine vector (ChAdOx1) from the Serum Institute of India (SII), Pune, India (in collaboration with the University of Oxford, U.K., and pharma giant, AstraZeneca) and an inactivated virus vaccine from the Bharat Biotech Ltd., Hyderabad, India (in collaboration with the National Institute of Virology of Indian Council of Medical Research (ICMR), India). Priority for vaccination was given to an estimated thirty million healthcare and frontline workers [2]. Experts in disease control, immunization, public health, and information technology recommended strategies to be employed in India’s COVID-19 vaccination program. According to the strategy for COVID-19 vaccination in India, the country with the second-highest population and cases of infected patients, two vaccines, AZD1222 and BBV152, were approved by the Drugs Controller General of India (DCGI), based on results from clinical trials [3].

In India, the first round of vaccination was administered to current healthcare workers, and the second round to priority groups, such as the elderly population, people with comorbidities, pregnant women, and children. Vaccination of people older than 60 years and those older than 45 years with comorbidities began on 1 March 2021 [4].

In an earlier study, researchers evaluated the diagnostic performance of seven rapid IgG/IgM tests and the EUROIMMUN IgA/IgG ELISA for antibodies against SARS-CoV-2 in patients with COVID-19. Their results demonstrated that commercial automated assays and ELISAs are suitable for the detection of IgG and total Ig antibodies against SARS-CoV-2 [5]. The present study mainly focused on evaluating post-vaccination immune responses in healthy individuals.

This study aimed to determine immune response and seroconversion after two doses of the recommended vaccines in a study group of healthcare workers by estimating the total immunoglobulin specific to the spike protein of SARS-CoV-2. Based on the literature on the methodology for the quantitative estimation of total immunoglobulin, we chose enzyme-linked immunosorbent assay (ELISA) to estimate the level of antibodies against the recombinant spike protein of SARS-CoV-2 [6,7,8].

## 2. Materials and Methods

### 2.1. Study Institutions

This study was conducted at the All India Institute of Medical Sciences Mangalagiri (AIIMS, M.G.), Andhra Pradesh, in collaboration with the SIBAR Institute of Dental Sciences, Guntur, Andhra Pradesh.

### 2.2. Study Design and Participants

This was a prospective cohort study.

### 2.3. Study Cohorts

For group 1, we recruited adult healthcare workers aged >18 years who received the AZD1222 vaccine at the COVID-19 vaccination center at AIIMS, M.G. The AstraZeneca AZD1222 vaccine was recommended by the government of India for patients aged ≥18 years. As per the recommended schedule, 2 doses (0.5 mL each) of the vaccine were administered at an interval of 4 weeks to all study participants of one group [9]. The first dose of both vaccines was administered to the study groups of healthcare workers on 18 January 2021. The immune response to the vaccine was measured 4 weeks after administration of the first and second doses, and then 24 weeks after the second dose, to verify the rate of seroconversion. This study cohort consisted of 88 participants (N = 88).

For Group 2, we recruited adult healthcare workers aged >18 years who received the BBV152 vaccine at the COVID-19 vaccination center at the SIBAR Institute of Dental Sciences, Guntur, Andhra Pradesh. The BBV152 vaccine was developed in India by Bharat Biotech, in collaboration with the Indian Council of Medical Research (ICMR)—National Institute of Virology (NIV). This group received the second dose of the BBV152 vaccine 28 days after the first dose, as per the recommendations [9].

Seropositivity to the vaccine was measured at 4 weeks after administration of the first and second doses and 24 weeks after the second dose to verify the rate of seroconversion. This study cohort consisted of 45 participants (N = 45).

A total of 133 HCWs who, reportedly, did not have a previous COVID-19 infection—that is, who were SARS-CoV-2 naïve—were enrolled into the study. HCWs on steroids, severe comorbid illnesses, children, and pregnant women were excluded from the study.

Data collection for this study began 6 February 2021 and ended 20 August 2021.

### 2.4. Study Group for Cut-Off Value Determination for ELISA Testing

Twenty-five healthy volunteers who had never been exposed to or immunized against COVID-19 were also enrolled to confirm the optical density (450 nm) 0.02, with the standard high control (units/mL) of “0” stated in the test kit manufacturer’s instruction manual.

As per the Indian Council of Medical Research (ICMR)’s COVID-19 testing guidelines, twenty-five healthy patients were not tested, since they lacked COVID-19 symptoms, and 5 mL whole blood samples were collected from these patients to determine the cut-off value for the total SARS-CoV-2 spike antibody level.

### 2.5. Blood Samples and Biosafety 

Strict infection control measures were taken during blood collection, and complete personal protective equipment (PPE) was worn by all personnel involved in the study during collection and processing of samples and testing in the biosafety level 2 (BSL2) laboratory [10].

Whole blood samples (5 mL) were collected in plain vacutainer rapid serum tubes from each participant 4 weeks after the first dose of vaccination. During the follow-up period, a second blood sample was collected 4 weeks after the second dose of vaccination and again at 24 weeks after the second dose. 

The serum was separated from whole blood immediately after collection and stored in separate aliquots at −20 °C in a deep freezer until further testing was performed in batches.

### 2.6. Antibody Measurement by Ig Total ELISA

The Human SARS-CoV-2 Spike (trimer) Ig Total ELISA Kit (Invitrogen-BMS2323, ThermoFisher Scientific Inc., Vienna, Austria) was used to detect the total Ig level in serum samples. The test was a solid-phase sandwich ELISA, which was designed to detect and quantify the level of total Ig against human SARS-CoV-2 in the serum. 

Trimerized spike protein was pre-coated in the wells of the supplied microplate. Samples and controls, including a high control, were used as a standard; these were then added to these wells and bound to the immobilized (capture) spike protein. The wells were washed, and secondary Ig antibodies conjugated to horseradish peroxidase (HRP) were added and bound to the primary antibodies. The wells were washed, and a substrate solution was added to react with the enzyme complex to produce a measurable signal. The total Ig antibody assay recognizes IgG, IgM, and IgA. The intensity of this signal is directly proportional to the concentration of the antibodies present in the original specimen.

Each ELISA run was performed with standards and controls in duplicates. The absorbance was measured using a BioTek Epoch2 microplate reader (BioTek Instruments, Inc., Winooski, VT, USA). Standard curves were plotted using four parametric log–log curves fitted via Gen5^TM^ v3.11 Microplate Reader and Imager Software (BioTek Instruments, Inc., Winooski, VT, USA). The background absorbance was subtracted from all data points, including the standards, unknown serum samples, and controls, prior to plotting. The concentrations of unknown serum samples and controls were determined using the standard curve (four parameter logistic (4PL) regression). We read the absorbance at 450 nm and determined the results of the assay quantitatively, as per the kit instructions over the range of 0 to 4000 units/mL total human SARS-CoV-2 Ig.

To set the cut-off value for results analysis, we have used change-point analyses [11]. Therefore, the cut-off antibody level was determined using the following formula:

Cut-off = Mean + 3 times the standard deviation

Based on the above calculation, a total SARS-CoV-2 spike antibody titer greater than the cut-off value of 907.58 U/mL was considered seropositive and confirmed seroconversion. 

An additional third sample was collected from participants in both groups who did not demonstrate any detectable level of total spike antibody or whose antibody level was below the calculated cut-off value in the samples collected 4 weeks after the second dose.

### 2.7. Statistical Analysis

The data were entered into a Microsoft Excel (MS Excel) spreadsheet and were analyzed using Epi-Info, IBM SPSS, and Open Epi software. The primary outcome of interest was seroconversion. The immune response to the vaccine—that is, seropositivity—was treated as a nominal variable with the following categories: seroconverted (yes) and seroconverted (no) with antibody titers more than 907.58 U/mL and less than 907.58 U/mL, respectively. The key variables of interest were the immune response to each vaccine, AZD1222 and BBV152, 4 weeks after the first and the second dose and 24 weeks after the second dose, in individuals who did not have prior history of COVID-19. 

The chi-square test and Fisher’s exact test were used to test the statistical significance of the association between two categorical variables. In this cohort study, the odds ratio (OR) and relative risk (RR) with corresponding 95% confidence intervals (CI) were calculated.

The geometric mean titer (GMT) and standard deviation (SD) with 95% CI were calculated for the antibody titers four weeks after the first and second doses of AZD1222 and BBV152 vaccines. An independent sample t-test was applied to determine whether there was any significant difference in GMTs of AZD1222 and BBV152 vaccines four weeks after the first and second doses.

A difference was considered statistically significant if the *p*-value was less than or equal to 0.05.

## 3. Results

Table 1 presents the demographic characteristics of the study participants. The AZD1222 vaccination group consisted of 57.9% (51/88) males and 42.1% (37/88) females. In the BBV152 vaccination group, 46.7% (21/45) of the participants were males and 53.3% (24/45) were females. The age range was 18–56 years (median 34 years) in the AZD1222 group and 19–52 years (median 26 years) in the BBV152 group. The mean age of participants was 33.1 and 29.8 years for the receivers of the AZD1222 and BBV152 vaccines, respectively.

As seen in Table 2, 71 out of 88 individuals (80.7%) had seroconversion from the AZD1222 vaccine 4 weeks after the first dose, whereas only 13 out of 45 individuals (28.9%) had seroconversion from the BBV152 vaccine 4 weeks after the first dose, and the difference was found to be highly statistically significant. Four weeks after the first dose, the seroconversion from the AZD1222 vaccine was 10.3 times (95% CI for odds ratio: 4.46–23.67) greater than that from BBV152. Meanwhile, 79 out of 88 individuals (89.8%) had seroconversion from the AZD1222 vaccine 4 weeks after the second dose, whereas only 16 out of 45 individuals (35.6%) had seroconversion from BBV152 vaccine 4 weeks after the second dose, and the difference was highly statistically significant. Four weeks after administration of the second dose, the seroconversion from the AZD1222 vaccine was 15.9 times (95% CI for odds ratio: 6.33–39.96) greater than that from BBV152 vaccine.

As shown in Table 3, the GMT (and SD) of the antibody titer (U/mL) measured 4 weeks after the first dose of AZD1222 and BBV152 vaccines were 6392.93 (4.92) and 1480.47 (9.32), respectively. An independent samples *t*-test was applied, and the difference was found to be highly statistically significant. The GMT (and SD) of the antibody titer (U/mL) measured 4 weeks after the second dose of AZD1222 and BBV152 vaccines were 6398.82 (3.24) and 990.38 (5.26), respectively. An independent samples *t*-test was applied, and this difference was found to be highly statistically significant.

Relative risk = 0.71, 95% CI for relative risk= (0.20, 2.49). As seen in Table 4, in total, 3 out of 19 individuals (15.8%) and 6 out of 27 individuals (22.2%) had seroconversion from the AZD1222 and BBV152 vaccine, respectively, 24 weeks after the second dose, but the difference was not statistically significant.

## 4. Discussion

The World Health Organization (WHO) accelerated the development of COVID-19 vaccines worldwide at the beginning of 2020 in order to control the COVID-19 pandemic. Among several vaccine preparations, the recombinant coronavirus spike (S) protein, a replication-deficient simian adenoviral vaccine (ChAdOx1-S), and a BBV152 inactivated viral vaccine were recommended by WHO [12]. The present cohort study evaluated the immune response of healthcare workers against SARS-CoV-2 spike protein by estimating the total Ig titer after the administration of AZD1222 and BBV152 vaccines. We found that the seropositivity rate four weeks after the first dose was significantly higher in AZD1222 than in BBV152 vaccine recipients.

Furthermore, we also found that the seropositivity rate four weeks after the second dose was significantly higher in AZD1222 vaccine vs. BBV152 vaccine recipients. Our study findings are similar to those of Singh et al. (2021), who found that the seropositivity rate of antispike antibody was significantly higher in Covishield (AZD1222 vaccine) vs. Covaxin (BBV152 vaccine) recipients (98.1 vs. 80.0%; *p* < 0.001) [13].

We also found that the geometric mean (and SD) of the antibody titer measured 4 weeks after the first and second doses of AZD1222 and BBV152 vaccines was significantly higher with the AZD1222 than with the BBV152 vaccine. A study by Singh et al. (2021) found that, after the administration of a second dose of Covaxin, the GMTs with 95% CI and median (IQR) antispike antibody titer only increased significantly after two doses. In contrast, the Covishield vaccine showed a greater than threefold increase in the antispike antibody GMT, even after a single dose.

However, the seropositivity rates 24 weeks after the second dose of AZD1222 and BBV152 vaccines did not show any statistically significant difference. Individuals who were infected with SARS-CoV-2 prior to receiving the AZD1222 and BBV152 vaccines did not report a significantly different seropositivity rate 4 weeks after the first and second doses of the AZD1222 and BBV152 vaccines.

A study revealed that, in the first phase (1/2) of a UK clinical trial testing two doses of the ChAdOx1 nCoV-19 (Covishield) vaccine, there was a significant increase in antispike IgG antibody after 56 days compared to its median titer after 28 days [14]. Voysey et al. (2021) reported the clinical efficacy of the above vaccine in phase 2/3 of trials conducted in the UK and Brazil and found that this vaccine was effective against symptomatic COVID-19 [15,16]. Indeed, the numerous freely available basic research data on the mechanisms of SARS-CoV-2 infection have convinced most developers of innovative vaccines to focus their efforts on inducing an immune response against the spike protein. The S protein of SARS-CoV-2 is the most suitable antigen for inducing neutralizing antibodies against the pathogen [17]. In one such study, the researchers evaluated antispike protein receptor-binding domain (S-RBD) antibodies, which represent a useful means of estimating the individual protection against SARS-CoV-2 infection, and observed a significant decrease in anti-RBD IgG levels within a short period following a complete two-dose vaccination cycle [18].

We have not estimated the antibody level in the study groups before vaccination, because the virus-specific neutralizing antibodies (NAbs), induced through either infection or vaccination, have the ability to block viral infection. The level of NAbs has been used as the gold standard to evaluate the efficacy of vaccines against smallpox, polio, and influenza viruses [19]. The rationale behind measuring antibodies before vaccination is because previous infection is associated with a stronger immune response after vaccination [20].

Various studies have reported increasing levels of neutralizing antibodies of IgM and IgG to SARS-CoV-2 at different time intervals. Long et al. (2020) reported that, in 285 patients with COVID-19 infection, there were acute antibody responses to SARS-CoV-2 [21]. Within 19 days, one hundred percent of the patients were positive for Immunoglobulin-G (IgG). The seroconversion of IgG and IgM occurred either simultaneously or sequentially. In addition, after symptom onset, the proportion of patients with virus-specific IgG positivity reached 100% at approximately 17–19 days. Virus-specific IgG and IgM antibody titers increased during the first 3 weeks after symptom onset.

There are many different vaccines prepared using different platforms, including recombinant vectors, DNA, mRNA in lipid nanoparticles, inactivated viruses, live attenuated viruses, and protein subunits against SARS-CoV-2 [22]. India had approved AZD1222/Covishield, a monovalent vaccine, and BBV152/COVAXIN, a whole virion-inactivated SARS-CoV-2 vaccine [23].

Based on immunogenicity studies in humans, the rate of seroconversion (a four-fold or greater increase over baseline) to S-binding antibodies was over 98% and 99%, 28 days after the first and second dose, respectively, in participants who were seronegative at baseline. The rate of seroconversion, as measured in a live neutralization assay, was over 80% and over 99%, 28 days after the first and the second dose, respectively, in participants who were seronegative at baseline [24].

The SARS-CoV-2 spike protein gene is expressed in the AZD1222 vaccine [25] and instructs the host cells to produce the S-antigen protein, which is unique to SARS-CoV-2. This allows the body to mount an immune response, and this information is retained in memory immune cells. The results of a clinical trial based on a median follow-up of 80 days showed that the efficacy of two doses of vaccine was 63.1%, irrespective of the interval between the doses. Efficacy tended to be higher when the interval was longer. The data reviewed at this time support the conclusion that the known and potential benefits of the AZD1222-S/nCoV-19 (recombinant) vaccine outweigh the risks [26].

The present study estimated antispike immunoglobulin levels in vaccinated populations to evaluate the immune response to vaccines as a convenient tool for assessing immunological response. This has been supported by a study conducted by researchers on the long-term persistence of SARSCoV-2 spike protein-specific and neutralizing antibodies in recovered COVID-19 patients. Their findings corroborate the reliability of estimating the antispike immunoglobulin levels as a convenient tool for assessing the immunological response of COVID-19-infected individuals to quantify the immunogenicity of the vaccines and therapeutic effects [27].

Finally, massive vaccination campaigns are expected to elucidate several critical aspects of the SARS-CoV-2 immunity. As larger populations get vaccinated, the durability of the immunity induced by the different vaccine strategies, as well as the finer details of the immune responses elicited, will emerge, including in those individuals with suboptimal immunity [28].

The limitations of this study included the small number of participants due to the high cost of the test kit and insufficient funds to support the testing of a larger number of participants. The lack of baseline antispike (S) serology is also a limitation of the study.

## 5. Conclusions

We found that the seropositivity response was significantly better in the AZD1222 group than in the BBV152 group, 4 weeks after the second vaccine dose, in 133 HCWs. Seropositivity response was also improved 24 weeks after the second dose in the BBV152 group compared to the AZD1222 group amongst 46 HCWs, but the difference was not statistically significant. This study found a low seroconversion rate in the study group vaccinated with the inactivated viral vaccine compared to the live vaccine.

## Figures and Tables

**Table 1:** Demographic data of the participants in this study.

Participants	Characteristics	Vaccine	
		AZD1222 N = 88	BBV152 N = 45
Age, years	Range	18.00–56.00	19.00–52.00
	Mean age (SD)	33.1 (6.7)	29.8 (9.4)
	Median age (IQR)	34 (29.00–37.00)	26 (22–36)
Age, years	<20 (N, %)	2 (2.3)	1 (2.2)
	20–29	22 (25)	25 (55.6)
	30–39	53 (60.2)	11 (24.4)
	>40 (N, %)	11 (12.5)	8 (17.8)
Gender	Male (N, %)	51 (57.9)	21 (46.7)
	Female (N, %)	37 (42.1)	24 (53.3)

Abbreviations: SD: standard deviation; IQR: interquartile range.

**Table 2:** Comparison of seroconversion rate 4 weeks after the first dose and the second dose of AZD1222 and BBV152 vaccines in SARS-CoV-2 naïve participants (N = 133).

Characteristics	SARS-CoV-2 Naïve Participant #		Odds Ratio, [95% CI], *p* Value	Relative Risk
	AZD1222 Vaccine	BBV152 Vaccine		[95% CI]
4 weeks after the first dose Seropositivity rate (%)	71/88, (80.7)	13/45, (28.9)	10.28 [4.46, 23.67] * p < 0.0001	2.79 [1.75, 4.47]
4 weeks after the second dose Seropositivity rate (%)	79/88, (89.8)	16/45, (35.6)	15.91[6.33, 39.96] * p < 0.0001	2.53 [1.69, 3.77]

# May include asymptomatic COVID-19 participants. * 95% CI for odds ratio calculated by the Wald method using WINPEPI software.

**Table 3:** Geometric mean (and SD) antibody titer after the first and second dose of AZD1222 and BBV152 vaccines in SARS-CoV-2 naïve participants.

Characteristics	SARS-CoV-2 Naïve Participant #		*p* Value
	AZD1222 Vaccine, N = 84	BBV152 Vaccine, N = 17 (Four Weeks after First Dose) N = 25 (Four Weeks after Second Dose)	
	Antibody Titer, Geometric Mean (SD) [95% CI], in U/mL	Antibody Titer, Geometric Mean (SD) [95% CI], in U/mL	
4 weeks after the first dose	6392.93 (4.92)	1480.47 (9.32)	0.0001
	[6391.88–6393.98]	[1476.04–1484.9]	
4 weeks after the second dose	6398.82 (3.24)	990.38 (5.26)	0.0013
	[6398.13–6399.51]	[988.31–992.44]	

# May include asymptomatic participants with COVID-19. Differences were considered statistically significant at *p*-value < 0.05.

**Table 4:** Seroconversion from the AZD1222 and BBV152 vaccines 24 weeks after the second dose (N = 46) without previous COVID-19 infection.

Immune Response to Vaccine Twenty-Four Weeks after the Second Dose of Vaccine	Seroconverted	
	Yes	No
AZD1222	3 (15.8%)	16 (84.2%)
BBV152	6 (22.2%)	21 (77.8%)

Fisher exact test, *p* = 0.88. Odds ratio = 0.66, 95% CI for odds ratio by the Wald method = (0.14, 3.03).

## Data Availability

Not applicable.
